# Effects of spatial confinement on migratory properties of Dictyostelium discoideum cells

**DOI:** 10.1080/19420889.2021.1872917

**Published:** 2021-01-28

**Authors:** Yuri Belotti, David McGloin, Cornelis J. Weijer

**Affiliations:** a School of Science and Engineering, University of Dundee, Dundee, Scotland, UK; b School of Life Sciences, University of Dundee, Dundee, Scotland, UK

**Keywords:** Chemotaxis, cell-migration, cell-polarization, blebbing, actin-myosin cytoskeleton, confinement, microfluidics

## Abstract

Migratory environments of various eukaryotic cells, such as amoeba, leukocytes and cancer cells, typically involve spatial confinement. Numerous studies have recently emerged, aimed to develop experimental platforms that better recapitulate the characteristics of the cellular microenvironment. Using microfluidic technologies, we show that increasing confinement of Dictyostelium discoideum cells into narrower micro-channels resulted in a significant change in the mode of migration and associated arrangement of the actomyosin cytoskeleton. We observed that cells tended to migrate at constant speed, the magnitude of which was dependent on the size of the channels, as was the locomotory strategy adopted by each cell. Two different migration modes were observed, pseudopod-based and bleb-based migration, with bleb based migration being more frequent with increasing confinement and leading to slower migration. Beside the migration mode, we found that the major determinants of cell speed are its protrusion rate, the amount of F-actin at its leading edge and the number of actin foci. Our results highlighted the impact of the microenvironments on cell behavior. Furthermore, we developed a novel quantitative movement analysis platform for mono-dimensional cell migration that allows for standardization and simplification of the experimental conditions and aids investigation of the complex and dynamic processes occurring at the single-cell level.

## Introduction

Cell migration plays a pivotal role in a variety of biological processes ranging from embryogenesis to wound healing [1] as well as in some pathological processes such as chronic inflammatory diseases, osteoporosis, and tumor metastasis [2]. Most cancer, epithelial and mesenchymal cells have been demonstrated to effectively navigate through tissues through matrix proteolysis [3]. On the other hand, amoeboid cells such as leukocytes adopt nondestructive migratory strategies [4] that opened up questions about how these cells can efficiently and quickly navigate through dense tissues. How cells interpret complex and dynamic external stimuli and actuate the internal cytoskeletal machinery to achieve motion is still the focus of active investigation [5]. Numerous cell types can sense a variety of biochemical and physical cues. For instance, during *chemotaxis*, cells actuate a directed migration as a consequence of directional sensing of chemical concentration gradients [6].

Dictyostelium discoideum (Dd) is a leading model for the study of eukaryotic chemotaxis [7,8]. Research conducted on this organism contributed to generate data that anticipated results lately found in other organisms and enabled the establishment of experimental methods successively used in other systems. Eukaryotic cells such as Dictyostelium and neutrophils exhibit extraordinary sensitivity to external chemical gradients [9]. The interactions between signaling, regulatory and cytoskeletal components involved in gradient sensing are conserved between Dd and neutrophils (similarities and differences between the two organisms are reviewed in [10,11] and [7]. Chemotaxis plays a key role in the life cycle of Dd. Upon binding between the cyclic adenosine monophosphate (cAMP) molecules and their receptors cAR1, which are situated on the cell’s surface, signal transduction cascades are activated and result in a local, dynamic modification of the cytoskeleton leading to the polarization of the cell and its motion toward an increasing concentration of cAMP [6,12]. The key aspects of the cell polarization are: (1) the polymerization of actin filaments at the leading edge of the cell that are associated with the extension of pseudopodia [10], (2) the assembly of myosin II (myo-II) at the trailing edge that is involved in the contraction of the uropod [13]. However, the mechanisms by which signal sensing, cell polarization and movement are coupled remain unresolved [14].

Most of the studies on the mechanisms governing cell migration arose from *in vitro* investigations on planar surfaces, but recent studies showed that this classical picture of cell locomotion is inadequate to recapitulate the properties of cell migration within tissues [15]. Recent experiments have shown striking different migration behaviors of cells confined in constrained microenvironment: motility characteristics, such as the velocity, cell adhesions and locomotory strategy can be very different in an environment with different dimensionality [16–19].

In this paper, we developed a migration assay that allows for spatial confinement of Dd cells while exposing them to a linear gradient of cAMP. This platform was designed to confine cells in environments with only one degree of freedom to simplifying the complexity of the actual dynamic, three-dimensional motion of migrating cells. In this way, we were able to investigate how the cytoskeleton responded to such simplified boundary conditions and this allowed us to better understand how the main cytoskeletal components are orchestrated to achieve a directed migration. Specifically, we focussed on investigating the response of the acto-myosin cytoskeleton to confined environments and how this relates to the migration properties.

## Results

### Actomyosin cytoskeleton in spatially confined cells

**Figure 1.**
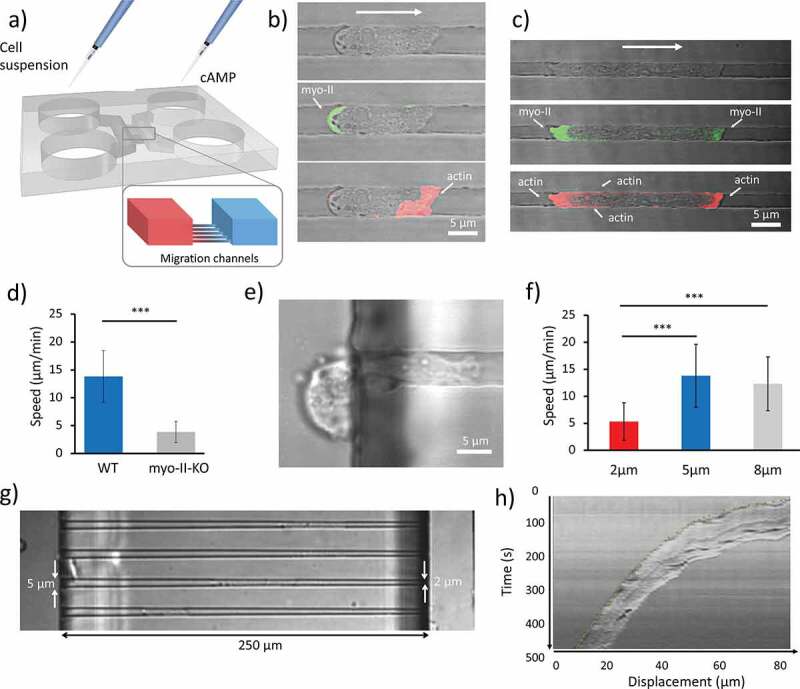
Effect of spatial confinement on cell migration. (a) 3D rendering of the microfluidic device. The two symmetric loading arms are connected by migration channels arranged in ’ladder-like’ structure. Cell suspensions are loaded on one side and cAMP on the opposite side. A set of migration channels bridges the two arms in which a cAMP gradient is established by diffusion. (b, c) Sets of images showing a cell migrating in a 2 μm x 5 μm and 2 μm x 2 μm channels, respectively. In the middle and lower figures, the localization of myo-II and F-actin is indicated, respectively. Bright-field and confocal images are merged. (d) Mean velocities of WT cells (n = 56, analyzed over 11 independent experiments) moving in 5 μm wide, 2 μm high channels and mhcA^−^ cells (n = 30, collected in 11 independent experiments) confined in 5 μm wide, 3.5 μm high channels. (e) Representative mhcA^−^ cell stuck at the entrance or a 2 μm high, 5 μm wide channel. On the right-end side, the leading edge of the cell elongates toward higher concentration of cAMP. On the left, the uropod is blocked outside the migration channel. (f) The velocity of cells measured in micro-channels with different width. The histogram shows the average speed calculated over 48 cells analyzed over 14 independent experiments, 56 cells analyzed over 11 independent experiments, and 56 cells analyzed over 9 independent experiments, respectively. The height of all channels is 2 μm. Error bars indicate SD. ***p < 0.001. (g) Cells migrating in tapered channels. (h) Representative kymograph taken along the channel’s axis of a WT cell migrating in a tapered channel. The picture shows that the speed decreases constantly over time as a consequence of the linear decrease in channel width

First, we sought to investigate how the actomyosin cytoskeleton responds to chemotactic cues in under spatial confinement. Dd cells were loaded into the ladder-like microfluidic device where cells and chemoattractant could simply be added and controlled by adjusting the volume of the media in each loading reservoir, without the use of syringe or pressure pumps (Figure 1(a)). Then, we qualitatively analyzed the localization of Myosin-II and F-actin by using cells where the two proteins were labeled with GFP and RFP-LifeAct, respectively. Furthermore, we quantitatively tested the behavior of wild type (WT) and myosin-II heavy chain knockout cells (mhcA-), where the complete coding sequence of the heavy chain was deleted and which display deficient contraction and cortical rigididty [20]. The localization of actin and Myosin II for a cell in a 5 μm wide channel is shown in Figure 1(b). As expected, the myo-II (in green) localizes at the uropod of the cell, while the actin is abundant at the cell front. This type of localization is similar to that of cells migrating in 2D on a substrate. Interestingly, when the width of the channels is reduced to 2 μm by 2 μm, actin shows an increased localization at the uropod and the plasma membrane, in areas where the cell is in contact with the walls of the channel (as shown in Figure 1(c) and movie S1), whereas myo-II exhibited an increased localization at the pseudopod, as shown in Figure 1(c) and Movie S2. The role of myo-II in confined migration was investigated using mhcA^−^ cells. These cells exhibited significant slower migration (3.9 ± 2 μm/min; mean ± SD) compared with the WT (13.8 ± 5.8 μm/min; mean ± SD), as shown in Figure 1(d). It is worth noting that in the case of the mhcA^−^ cells, we needed to increase the height of the migration channels to 3.5 μm as cells did not manage to fully penetrate the 2 μm high channels, since the nucleus was stuck in the back of the cell and could not enter the channel, as shown in Figure 1(e) and Movie S3

### Relationship between spatial confinement and cell speed

**Figure 2.**
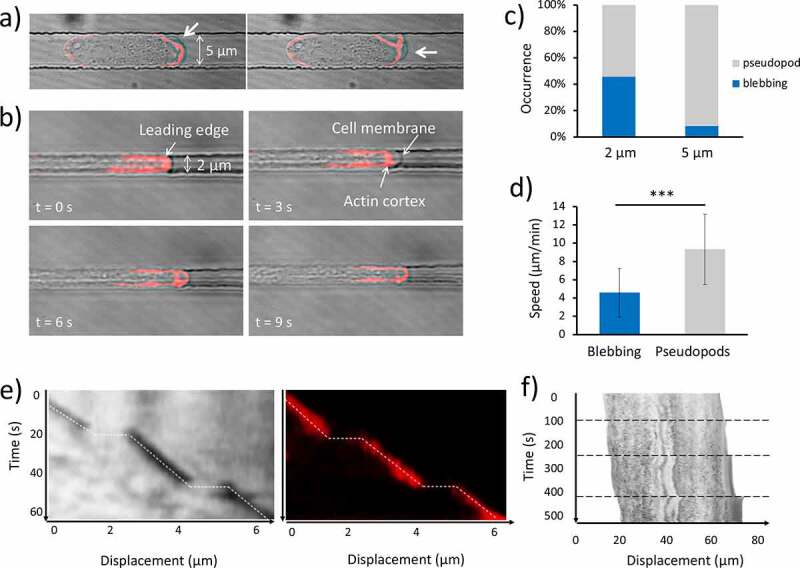
Effect of the spatial confinement on migration mode. (a) Consecutive time-lapse images of a cell moving in 2 μm x 5 μm channel in which F-actin is labeled with RFP-LifeAct. Bright-field and confocal images are merged. In each frame, a bleb forms at a different position of the leading edge, as indicated by the white arrow. (b) Consecutive time-lapse images of a cell moving in 2 μm x 5 μm channel. At t = 0 s F-actin can be observed at the leading edge; at t = 3 s the membrane expands quickly without being supported by the actin cortex; at t = 6 s cortical actin starts to translocate inside the bleb; at t = 9 s the cortex at the leading edge is restored to the normal conditions. (c) Percentage occurrence of the two migration modes as a function of the size of the microchannels. (d) Speed of cells that migrate by “blebbing mode” (N = 17, collected in 13 independent experiments) and “pseudopod mode” (N = 14, collected in 13 independent experiments). Mean value is shown. Error bars indicate the SD. ***p < 0.001. (e) Kymographs taken at the middle line of the migration channel of the blebbing cell in (b). The pictures show only a small portion of the leading edge of the cell. The position of the membrane is outlined by the broken line in both the bright field and the confocal images. The horizontal segments correspond to the bleb generation during which the membrane is detached from the cell cortex and extended impulsively forward without being supported by actin. (f) Kymographs from the middle line of a blebbing cell. Horizontal broken lines delineate the bleb formation event. The clearer stripe in the middle of the cell corresponds to the nucleus

To assess how the spatial confinement affects the migration velocity we developed a microfluidic device with migration channels of different widths: 2, 5 and 8 μm, whereas the height was constant and equal to 2 μm. The results of the quantitative analysis presented in Figure 2(f) show that cells that migrate in 2 μm wide channels are significantly slower (5.3 ± 3.5 μm/min; mean ± SD) than those that migrate in 5 and 8 μm wide channels (13.8 ± 5.8 μm/min and 12.3 ± 4.9 μm/min, respectively; mean ± SD). Dd cells have been reported to move on a flat surface without spatial confinement at an average speed of 11.7 ± 1.4 μm/min toward the tip of a micropipette that releases cAMP [21]. These values are comparable with those of cells confined in 5 and 8 μm wide channels as shown in Figure 1(f). Notably, when moving in 2 μm wide channels, cells exhibited a slower migration phenotype. To further investigate this behavior, we developed a variant of the chip that allowed us to expose each cell to a progressively decreasing width between 5 and 2 μm across the 250 μm long channels, as shown in Figure 1(g). Most of the cells analyzed (> 90%) showed a velocity profile similar to that shown in Figure 1(h).

### Relationship between spatial confinement, actin foci and migratory modes

When cells migrated through microchannels two distinct ways of motion were observed: 1) the pseudopod-based migration mode and 2) the blebbing mode. In the first case, the protrusions were irregular in shape and appeared enriched in F-actin. In the second case, the protrusions were much faster, almost spherical and devoid of F-actin at the early stage of their formation. In all our experiments, we observed that each migration mode tended to be persistent during the journey of each cell through the microchannels. A detailed example of the actin dynamics during bleb generation is depicted in Figure 2(a,b). The kymograph taken from the axis of the channel of the blebbing cell in Figure 2(b) is shown in Figure 2(e). The two kymographs show two consecutive blebbing events captured using the confocal and the brightfield images, respectively. The occurrence of a bleb corresponds to the horizontal segments, indicating that the time-scale of the bleb formation is below the acquisition time (3 s).

**Figure 3.**
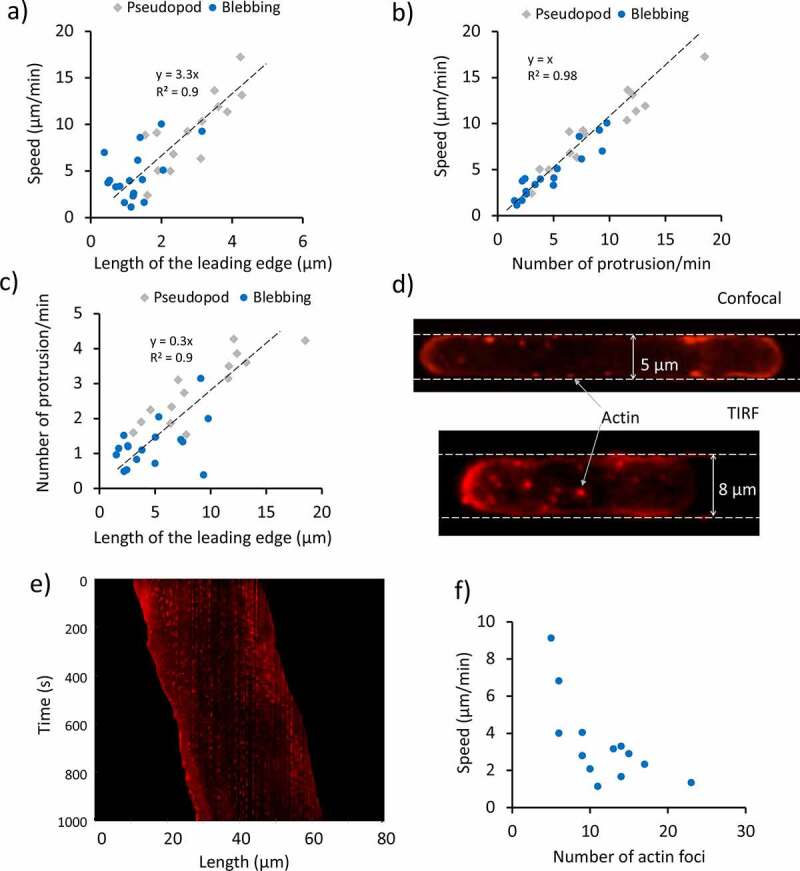
Effect of the migration mode and focal adhesions on the cell speed. (a) Relationship between the extension of the actin polymerization zone at the leading edge and the cell speed for 31 cells confined in 2 μm high, 2 μm wide channels, observed in 13 independent experiments. (b) Relationship between the mean velocity of the single cells and the protrusion rate for 31 cells confined in 2 μm high, 2 μm wide channels, observed in 13 independent experiments. (c) Relationship between the protrusion rate and length of the leading edge for 31 cells confined in 2 μm high, 2 μm wide channels, observed in 13 independent experiments. The cells in (a-c) are the same as in Figure 2(d). (d) Confocal and total internal reflection fluorescence (TIRF) images of actin foci. The confocal image shows a Dictyostelium cell migrating in a 5 μm by 2 μm channel, whereas the TIRF one shows a cell migrating in an 8 μm by 2 μm channel. In the latter case, only the foci in the ventral region are imaged. The cells are F-actin labeled (RFP-LifeAct). The red spots represent the actin foci. (e) Kymograph of an RFP-LifeAct-labeled cell imaged using a confocal microscope. Vertical broken lines indicate the periodic activation of actin foci forming in stationary positions throughout the migration. The kymograph is taken for a section of the cell close to the PDMS wall. (f) Relationship between the mean cell speed of migration and the mean number of foci for 12 cells analyzed in 6 independent experiments

Indeed, velocities of about 10 to 20 μm/s have been measured during the expansion phases [22]. Interestingly, the constant localization of F-actin at the leading edge is interrupted by the formation of the blebs. It can be noticed that cells alternate sudden displacements, and constant velocity motion, in a relatively periodic way. The conditions that lead to the formation of pseudopods rather than blebs are still not clear, but we observed that Dd cells exhibit an increasing tendency to protrude blebs when the size of the channel decreases, as shown in Figure 2(c). The histogram shows that 16 out of 35 cells migrating in 2 μm wide channel moved via blebbing motion, while only 3 out of the 36 cells in 5 μm wide channel adopted such a mode. On average, blebbing cells exhibit lower migration speed (4.6 ± 2.7 μm/min; mean ± SD) than cells moving by pseudopods (9.3 ± 4 μm/min; mean ± SD) as shown in Figure 2(d). Figure 2(f) shows a kymograph from the centreline of a blebbing cell moving in a 2 μm wide channel. It is clear that the rear contracts continuously while the front protrudes episodically. During bleb formation, it appears that the entire cell body is quickly shifted forward. It is important to note that a minority of cells exhibited a mixed phenotype where pseudopodia and blebs coexisted. In these cells, the localization of actin at the leading edge appeared at an intermediate level between the two modes. This suggests that a diminished presence of actin at the cell front is the main cause of the bleb formation. As we recently reported, the extension of the area in which the F-actin polymerization takes place varies from cell to cell, and it appeared to decrease as the channel cross-section decreased, impacting the cell speed [23]. This behavior is not only present in pseudopod-based moving cells, but also blebbing cells. A linear relationship between the cell speed and the length of the actin enriched front is shown in Figure 3(a) for 31 cells. More specifically, we considered only the length of this area, as the width is fixed by the width of the channel (2 μm). It appeared that blebbing cells tended to have a smaller actin-enriched leading edge and to move slower than the pseudopod-driven cells. Furthermore, the plot in Figure 3(e) shows that on average blebbing cells tend to move slower than the pseudopod-driven cells, due to a lower protrusion rate. Lastly, the relationship between the protrusion rate and the length of the leading edge is shown in Figure 3(f).

The use of F-actin labeling allowed us to investigate the spatial distribution of actin foci in spatially confined cells, as shown in Figure 3(d). These foci represent adhesive contacts between the cortical actin of Dictyostelium cells and the substratum, and have been observed and characterized in previous studies and sometimes but not always coincide with focal adhesion contacts [13,24–26]. The spatial confinement enabled us to investigate the temporal behavior of multiple foci at the same time using the kymograph analysis tool, as they tend to be aligned along the channel’s walls in confocal image sequences. The kymograph in Figure 3(e) was generated from a line in the proximity of a microchannel’s wall. The vertical broken lines in the kymograph indicate the activation and inactivation of actin foci. Interestingly, it could be noticed that their occurrence tends to be periodic, as also shown in Figure S3. It is important to note that in this work the term “periodic” has been used in the sense of “repetitive temporal pattern”, as in the previous literature of cell motility [22,27–29].

The distribution of the actin foci duration and period are shown in Figure S4. Both distributions could be fitted using a lognormal distribution, and the extrapolated averaged foci duration was about 13.8 ± 4.1 s (M ± SD), whereas their mean period was 32.7 ± 10.7 s (M ± SD). Another interesting observation came from the relationship between the mean cell speed and the number of foci. In fact, it appeared that cells tended to have a consistent number of foci over time. By plotting the mean migration speed against the mean number of foci for 12 cells, it became evident that the two quantities were inversely proportional, as shown in Figure 3(c).

## Discussion

In this paper, we showed how several properties of Dd cells are affected during their movement under spatial confinement. First, we sought to investigate how the actomyosin cytoskeleton was affected by increasing spatial confinement. We observed that an increased amount of F-actin is required in the vicinity of the walls of the channel (Figure 1(c)). Similar results have been reported for cancer cells of higher organisms: cells that undergo a transition into an amoeboid mode of migration in 3D collagen matrix show reduced accumulation of F-actin at the cell front [2]. Furthermore, we found that cell velocity depends on the degree of spatial confinement. We set the height of the channel to 2 μm, which is in the order of the typical diameter of the nucleus in Dictyostelium discoideum cells [30,31]. It turned out that a width of more than 8 μm was insufficient to laterally confine migrating of cells of average size (Movie S3). Cells were confined in 8, 5 and 2 μm wide channels, however the average cell speed was only significantly reduced in 2 μm wide channels. This was further investigated using tapered channels that enabled us to study the effect of a progressively decreasing width (from 5 to 2 μm) on each cell rather than comparing averaged information from different cells migrating in channels with a different cross-section. Velocity profiles similar to those described in Figure 1(e) have been shown to fit exponentially decaying curves [32]. Starving Dictyostelium cells have been reported to move on a flat surface at an average speed of 11.7 ± 1.4 μm/min toward the tip of a micropipette that releases cAMP [21]. Despite the absence of spatial constraint this speed is comparable with that of cells confined in 5 and 8 μm wide channels (Figure 1(f)).

Taken together our results suggest that the nucleus might play an important role in limiting the cell speed as the channel’s cross-section approaches the size of the nucleus. One likely explanation is that the nucleus, which is known to be the stiffest cellular organelle [33–35], pushes the cell’s cortex against the walls of the channel and glass substrate thus generating a significant friction force. The cell lengthens uniformly as it decelerates while it moves along the progressively narrower channel. The nucleus is not displaced toward the uropod as a consequence of the encountered friction due to the fact that migrating cells are able to maintain their nucleus in the proximity of the center of mass due to a microtubule-based centering mechanisms [36]. The loss of myo-II resulting in defective cell contractility, leads to a slower migration of these mutants with respect to WT cells, consistent with previous studies on Dictyostelium cells moving in 2D under-agar assays [13,37]. The observation that mhcA^−^ cells cannot enter 2 µm high channels can likely be ascribed a role for Myosin-II in mediating the required deformation of a rigid nucleus.

The existence of two different migration modes was demonstrated in Dictyostelium cells migrating in a confined 3D environment: an actin-independent blebbing motion, and an actin-based pseudopod motion in agreement with previous observations of blebbing under compression [38,39]. We did not observe any blebbing motion in the case of mhcA^−^ cells, confirming the importance of myo-II in the generation of blebs, already shown in previous studies conducted in 2D environments [40,41]. Interestingly, we observed that the nucleus is quickly pushed forward during bleb formation (as shown in Figure 2(f)). This is in line with previous work which showed that hydrostatic pressure is generated in the cytoplasm as a consequence of the myo-II-mediated contractions at the uropod [42–44]. Moreover, it has been shown that the synthetic enhancement of the contractility in cells that normally do not exhibit blebbing, resulted in bleb generation [45]. We also observed that blebbing tended to occur in a periodic fashion once confined in smaller microchannels. Similar observations have been made by Maugis et al. [22] where cells of the human parasite Entamoeba histolytica were aspirated in micropipettes. In general, these cells form blebs at a random rate, but when forced into the micropipettes, the blebs start to be protruded periodically. The authors suggested that myosin-driven cortical contractions would generate an instability of the intracellular pressure that, in the case of a constant geometry and shape of the environment, would induce a periodic formation of blebs. Although the cause of the occurrence of these migration modes is not fully understood, we showed that the blebbing motion is associated with a higher degree of confinement. The reduced abundance of F-actin at the leading edge could trigger the formation of blebs. This would explain why the two modes appeared to be mutually exclusive. This is supported by previous work by Langridge and Kay [40], where cells with a partially inactivated Arp2/3 complex exhibit a large increase in blebbing. The authors suggested that bleb formation is inhibited by F-actin. An alternative scenario could see blebs and pseudopodia as strongly correlated types of protrusions, which coexist in open space or 2D migration [46], and the spatial constriction would “select” the most efficient mode for each cell, depending on the relative amount of F-actin present at the leading edge.

In order for the blebs to contribute to the cellular motion in confinement, they need to be localized at the leading edge. The observed reduced amount of F-actin at the front can favor both local detachment of the plasma membrane from the cytoskeleton, as well as local rupture of the actin cortex. Both phenomena have been linked to the formation of polarized blebs [47]. Moreover, local contractions of the actomyosin cortex have also been associated with local increases in pressure that in turn could tear the membrane from the cortex, and result in bleb initiation [47]. The unusual increased localization of myo-II in the front of migrating cells that we observed in the case of high spatial confinement, therefore likely contributes to the increased blebbing observed. It will be of interest to establish what mechanism determines this unusual accumulation of Myosin II in the leading edge. The increases in the global pressure required for the cell content to flow through the thin 2 µm x 2 µm channel may not be achieved by Myosin II driven contraction at the back of the cell alone and may require graded contraction in the front, thereby playing a key role in bleb initiation, at the leading edge.

A big variability in the cells’ mean velocity was observed. This cell-to-cell variability can be explained as the result of multiple characteristics of an individual cell, its protrusion rate (consistent with what shown by Lombardi et al. [48]), the amount of F-actin at its leading edge and the mean number of actin foci. The latter result is consistent with the relationship between instantaneous velocity and the instantaneous number of foci by Uchida and Yumura [25]. Similar results have also been recently shown for Dictyostelium cells migrating in micro-channels in the absence of a chemoattractant [49].

Overall, our results highlight the impact of the microenvironment’s dimensionality on cell migration. Furthermore, our assay, combined with standard imaging techniques, allows standardization and simplification of the experimental conditions and aids quantitative investigation of the complex and dynamic processes occurring at the cell level.

## Methods

### Microfluidic device

The structure of the microfluidic device is similar to that used in previous studies [17,50]) and is characterized by a ladder-like structure where two “loading channels” are bridged by “migration channels”. The loading channels are connected to two cylindrical reservoirs that act as infinite source and as the sink, respectively. The three-dimensional (3D) schematics of the device are shown in Figure 1(a). The cell suspension is loaded in one side of the chip, whereas the other symmetric half is loaded with the chemoattractant (cAMP).

### Device fabrication

The design of the mold is carried out in QCAD professional (version 3.9.8.0). The molds were developed using a two-layer photolithographic techniques [23]. SU-8 2002 and SU-8 2075 were spin coated onto a 6-in silicon wafer (IDB Technologies) according to the required height of the film. High-resolution photomasks were used to transfer the features of each layer through ultraviolet (UV) illumination. The two layers were aligned using a mask aligner. Replicas of the patterned molds were obtained by soft lithography using polydimethylsiloxane (PDMS) (Sylgard 184 Silicone Elastomer Kit; Dow Corning Corporation). The PDMS base and curing agent were mixed at 10:1 ratio, degassed in a vacuum desiccator. After baking for 3 h at 80°C, the cured PDMS layer was demolded and ports were punched through the inlets and outlets regions using 8 mm biopsy punchers (Harris Uni-Core). Patterned PDMS was reversibly bonded to standard microscope glass slides (VWR) exploiting the Van der Waals forces that generate at the interface between the PDMS and the glass.

### Cell culture

Dd cells (strain Ax2) were grown in shaken suspension in HL5 medium using glass flasks at 22°C. Cells were harvested when the cultures reached ∼4 × 10^6^ cells/mL cells by centrifugation (400 × g for 2 min). Cells were washed twice and resuspended in KK2 buffer at the concentration of 1 × 10^7^ cells/mL and starved for 4.5 h while subjected to periodic stimulation with 10^−7^ M cAMP (47). Myosin II heavy chain knockout cells (mhcA-) were the whole protein coding sequence had been deleted obtained from Dicty Stock Center (DBS0238693). To prevent the cells from becoming multinucleate due to their cytokinesis defect, they were not grown in suspension culture but instead in 10 cm bacteriological plastic dishes in 10 ml of HL5 medium and harvested when 50% confluent, followed by starvation as described above. In these conditions the cells stay predominantly mononucleate, since they divide efficiently by traction mediated fission.

To visualize actin dynamics a strain transformed with Lifeact-red fluorescent protein (RFP) was used; to visualize myosin dynamics a myosin II heavy chain GFP N-terminal knock-in strain was used. The GPF knock-in strain was made using established homologous recombination techniques, using a five-amino-acid linker (GALVG) resulting in the GFP-myosin fusion sequence MDELYKGALVGNPIHDRT.

### Cell Loading

After starvation, cells were washed twice and suspended in KK2 at a final concentration of 1 × 10^7^ cells/mL. An aliquot of 200 μL cell suspension was loaded inside one of the two inlets of the microfluidic device using a pipette, while the opposite inlet was filled with 200 μL of cAMP (1 μM). A transient flow directed from the source to the sink reservoirs establishes and lasts until the hydrostatic pressure at these two ends of the loading channels is balanced. At the equilibrium, a linear spatial gradient of chemoattractant establishes inside the bridging channels, by diffusion, as previously shown by Abhyankar et al. [51]. Cells adhere to the substrate and those in the proximity of the entrance of the migration microchannels move inside the channels and migrate up the gradient. At the end of each experiment, the glass coverslip was separated from the PDMS chip and both surfaces were cleaned with 70% ethanol and stored for following experiments. Additionally, to maintain the chemical gradient steady, the four reservoirs were covered using small pieces of PDMS.

### Imaging, image analysis, and statistical analysis

Experiments were conducted at room temperature and cells imaged with a Leica SP2 AOBS confocal microscope equipped with either a 100x, 1.4 N.A. plan apochromat, or a 40x, 1 N.A. PL fluorotar objective. The inclusion criteria for our analysis were that cells had to travel directionally toward the higher end of the chemical gradient. In the case of trains of cells, only the first cell was considered. Data analysis was conducted in Excel for Mac 2011, Matlab R2018b. The velocity of the cells was estimated exploiting the fact that cells tended to exhibit a constant average velocity. Specifically, kymographs were generated from each migrating cell, at the location of the axis of the channels. The mean velocity <v> was then calculated using the following formula:

(1) $$<v\geq \;\frac{1}{tan\left(\alpha \right)}.\frac{pixel\;size}{time\;step}.60,$$

where α is the angle indicated in Figure S1, expressed in radians. The velocity calculated using Equation (1) gives the cell speed in μm/min.

To quantify the temporal evolution of actin foci, from each image sequence we generated two kymographs (one for each wall) from which we extracted the vertical size of each actin focus, that represents its duration, and the vertical distance between consecutive actin foci, that corresponds to the period of their activation. We then computed the mean period and the mean duration of the actin foci for different cells.

## Supplementary Material

Supplemental Material
